# Antiplasmodial Activities of Homogentisic Acid Derivative Protein Kinase Inhibitors Isolated from a Vanuatu Marine Sponge *Pseudoceratina* sp.

**DOI:** 10.3390/md7040640

**Published:** 2009-11-23

**Authors:** Nicolas Lebouvier, Valérie Jullian, Isabelle Desvignes, Séverine Maurel, Arnaud Parenty, Dominique Dorin-Semblat, Christian Doerig, Michel Sauvain, Dominique Laurent

**Affiliations:** 1 Laboratoire de Chimie, Université de la Nouvelle-Calédonie, BP R4, 98851 Nouméa cedex, New Caledonia; E-Mails: isabelle.desvignes@univ-montp2.fr (I.D.); arnaud.parenty@gmail.com (A.P.); 2 Laboratoire de Pharmacochimie des Substances Naturelles et Pharmacophores Redox, Université de Toulouse, UPS, UMR 152, 118, rte de Narbonne, F-31062 Toulouse cedex 9, France; E-Mails: jullian@cict.fr (V.J.); chevalleyseverine@yahoo.fr (S.M.); michel.sauvain@ird.fr (M.S.); dominique.laurent@ird.fr (D.L.); 3 Institut de Recherche pour le Développement (IRD); UMR 152, 118, rte de Narbonne, F-31062 Toulouse cedex 9, France; 4 INSERM U609, Global Health Institute, Ecole Polytechnique Fédérale de Lausanne, CH-1015 Lausanne, Switzerland; E-Mails: dominique.dorin@epfl.ch (D.D.-S.); christian.doerig@epfl.ch (C.D.); 5 Wellcome Centre for Molecular Parasitology, University of Glasgow, Glasgow G12 8TA, Scotland, UK

**Keywords:** Pseudoceratina, Pfnek-1, homogentisic acid derivatives, Plasmodium falciparum

## Abstract

As part of our search for new antimalarial drugs in South Pacific marine sponges, we have looked for inhibitors of Pfnek-1, a specific protein kinase of *Plasmodium falciparum*. On the basis of promising activity in a preliminary screening, the ethanolic crude extract of a new species of *Pseudoceratina* collected in Vanuatu was selected for further investigation. A bioassay-guided fractionation led to the isolation of a derivative of homogentisic acid [methyl (2,4-dibromo-3,6-dihydroxyphenyl)acetate, **4a**] which inhibited Pfnek-1 with an IC_50_ around 1.8 μM. This product was moderately active *in vitro* against a FcB1 *P. falciparum* strain (IC_50_ = 12 μM). From the same sponge, we isolated three known compounds [11,19-dideoxyfistularin-3 (**1**), 11-deoxyfistularin-3 (**2)** and dibromo-verongiaquinol (**3**)] which were inactive against Pfnek-1. Synthesis and biological evaluation of some derivatives of **4a** are reported.

## 1. Introduction

Malaria is a major health problem in tropical and subtropical regions and up to 2.5 million people die from it each year, mostly children in sub-Saharan Africa. *Plasmodium falciparum* causes the majority of deaths, and the spread of its resistance to many antimalarials (quinine, chloroquine, and mefloquine) has increased the need for development of new drugs. Natural products have provided clinically used antimalarials such as quinine and artemisinin and continue to make an important contribution to the discovery of new lead compounds. In past few years, not only plants but marine organisms have also been intensively investigated for obtaining new therapeutic agents against malaria [1–4].

As part of our search of new drugs against malaria, we have looked for inhibitors of a Pfnek-1 [5], a NIMA-related protein kinase of *Plasmodium falciparum,* in South Pacific marine sponges. This strategy previously led to the isolation of xestoquinone from *Xestospongia* sp. and to the characterization of its antiplasmodial activity [6]. An ethanolic crude extract from a new species of *Pseudoceratina* collected in Vanuatu was selected for its promising activity against Pfnek-1. Marine sponges of the order Verongida are characterized by tyrosine metabolites. Among them, sponges of the genus *Pseudoceratina* were the source of many bromotyrosine metabolites with interesting biological activities [7–18].

## 2. Results and Discussion

### 2.1. Chemistry

**Scheme 1.**
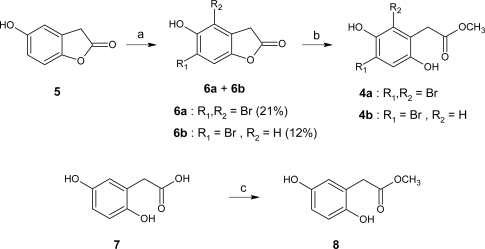
Synthesis of compound **4a**. *Reagents and conditions*: (a) *N*-bromosuccinimide, *p*-toluenesulfonic acid, CH_2_Cl_2_, 12 h, rt; (b) *p*toluenesulfonic acid, MeOH, rt, 24 h, 84% (**4a**), 78% (**4b**); (c) HCl_aq_ (1%), MeOH, rt, 24h, 97%.

The bromotyrosine metabolites, 11,19-dideoxyfistularin-3 (**1**)**,** 11-deoxyfistularin-3 (**2**) and dibromoverongiaquinol (**3**) were isolated from an ethanolic extract of *Pseudoceratina* sp. which was subjected to solvent partitioning between CH_2_Cl_2_ and H_2_O. The compounds were obtained from the CH_2_Cl_2_-soluble and -insoluble extracts by chromatography on silica gel and further purification by preparative thick layer chromatography or by reverse phase HPLC. The spectroscopic properties of the isolated bromotyrosine metabolites **1**–**3** were consistent with those previously published [19]. A bioassay-guided fractionation based on Pfnek-1 inhibition assay led us to isolate methyl (2,4-dibromo-3,6-dihydroxyphenyl) acetate (**4a**). An analogue of this compound, 4,6-dibromo-2,5-dihydroxyphenylacetic acid amide, was already isolated from the sponge *Verongia aurea* [20]. Isolation of **4a** from *Verongia aurea* was also briefly mentioned in a patent [21], but surprisingly, no spectroscopic data were reported. For the unambiguous identification of natural compound **4a** as responsible for activity against Pfnek-1, it was necessary to perform its synthesis, which was achieved as reported in Scheme 1.

The principal impediment of this synthesis was the difficulty in achieving the regioselective bromination of the phenyl ring. First attempts at bromination of methyl (2,5-dihydroxyphenyl)acetate (**8**) yielded a mixture of mono-, di- and tri-bromo derivatives. In order to increase the regioselectivity, we used as starting material 5-hydroxy-*3H*-benzofuran-2-one (**5**) which bears only one hydroxyl group. A similar pathway has been used for the synthesis of 4,6-dibromo-2,5-dihydroxyphenylacetic acid amide and derivatives [21,22]. Compounds **6a** and **6b** were thus obtained concurrently by bromination of **5** in the presence of *N*-bromosuccinimide and a catalytic amount of *p*-toluenesulfonic acid in CH_2_Cl_2_, and subsequently separated on silica gel by column chromatography. Ring opening of the benzofuran-2-ones was achieved with *p*-toluenesulfonic acid in MeOH to give **4a** and the monobrominated analogue **4b**. The spectroscopic properties (^1^H-, ^13^C-NMR, MS) of the synthesized compound **4a** were the same as those of the natural product, thus allowing its unambiguous identification (Table 1).

**Scheme 2.**
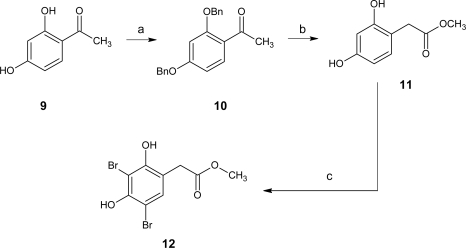
Synthesis of compound **12**. *Reagents and conditions*: (a) potassium carbonate, benzyl bromide, acetone, 15 h, rt, 73%; (b) perchloric acid, thallium trinitrate, THF/MeOH (1/5), 18 h, rt then palladium 10% on carbon, ammonium formate, MeOH, 4 h, rt, 51% two steps; (c) *N*-bromosuccinimide, *p*-toluenesulfonic acid, MeOH, 3 h, 40 °C, ultrasonic bath, 73%.

In order to study the influence of the position of the two hydroxyl groups and the ester chain on the phenyl ring, we synthesized an analogue **12** of the natural product with hydroxyl groups in the *meta* position (Scheme 2).

**Scheme 3.**
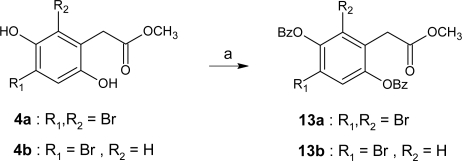
Synthesis of **13a** and **13b**. *Reagents and conditions*: (a) benzoyl chloride, trimethylamine, CH_2_Cl_2_, 1 h, 0 °C, 70%.

Treatment of 2′,4′-dihydroxyacetophenone (**9**) with potassium carbonate in the presence of benzyl chloride gave compound **10** in 73% yield. Willgerodt-Kindler rearrangement of **10** with thallium trinitrate and perchloric acid, followed by subsequent generation of hydroxyl groups in the presence of palladium 10% on carbon and ammonium formate gave compound **11**. Electrophilic ring dibromination was then carried out with *N*-bromosuccinimide and *p*-toluenesulfonic acid as catalyst in MeOH under sonication to obtain **12** in good yield. Finally, to study the substitution of the two hydroxyl groups, the benzoylations of compounds **4a** and **4b** gave the two lipophilic products **13a** and **13b** (Scheme 3).

Furthermore, the synthesis of **12** and **4a** allowed us to revise the structure of subreaphenol B, a natural compound isolated from the spon*ge Suberea mollis* and described as **12** [23]. However, the spectroscopic data (^1^H and ^13^C NMR) published for the natural compound did not match with our data for **12**, but rather with our data for **4a**, showing that the structure of subreaphenol B was identical to **4a** and not **12**.

### 2.2. Biological Properties

The ability of the pure natural products and of the synthetic compounds to inhibit Pfnek-1 activity was investigated (Table 2). Only compounds **4a** and **4b** exhibited a protein kinase inhibitor activity, particularly **4a** (IC_50_ = 1.8 μM) which was five times more active than **4b** (IC_50_ = 10 μM). 4,6-Dibromo-2,5-dihydroxyphenylacetic acid methyl ester (**4a**) was previously reported in a patent concerning homogentisic acid derivatives and their protein kinase C inhibitor activity [21]. The comparison of compounds **4a**, **4b** and **8** highlights the influence of the presence and number of bromine atoms on the phenyl ring. The non-brominated **8** was inactive, while monobrominated **4b** was moderately active; the dibrominated compound **4a** was the most active. Furthermore the presence of two free hydroxyl groups appears to be essential, as shown by: (i) the inactivity of the benzoyl analogues **13a** and **13b** and (ii) the inactivity of the lactone analogues **6a** and **6b**. In addition, the *para* position of the hydroxyl groups (hindered quinone system) is critical because the analogue **12** as well as the tyrosine metabolites **1**–**3** which have hydroxyl groups in the *meta* position are inactive. These results confirm the important role of quinone/phenolic part in the mode of action on Pfnek-1, a feature that is present in other Pfnek-1 inhibitors such as xestoquinone, halenaquinone, alisiaquinones A and B or alisiaquinol [6,24].

All the compounds were evaluated for *in vitro* antimalarial activity against a FcB1 *P. falciparum* strain (Table 2). Compounds **1**–**3** and **12** are inactive, while the other compounds have a weak antiplasmodial activity (12 μM < IC_50_ < 36 μM). Two homogentisic acid derivatives, methyl 2-(1′β-geranyl-5′β-hydroxy-2′-oxocyclohex-3-enyl)acetate and 2-(1′β-geranyl-5′β-hydroxy-2′-oxocyclohex-3′-enyl)acetic acid isolated from the leaves of *Glossocalyx brevipes* have already shown modest activities against *P. falciparum* [25]. The *para* position of the hydroxyl groups is essential to promote antiplasmodial activity, and also for Pfnek-1 activity (see above), suggesting that the cellular effect may be mediated by Pfnek-1. However, no other correlations can be established between the activity against Pfnek-1 and the inhibition of *P. falciparum* growth. A similar situation was observed with xestoquinone and alisaquinone. This suggests that there are other targets that are affected by the compounds, in addition to Pfnek-1.

Besides the activity of *Pseudoceratina* sp. extracts on Pfnek-1 and malaria parasites, strong antibacterial activities have also been observed. Activity was evaluated by the standard microdilution plate test with human pathogenic *Staphylococcus aureus* and *Escherichia coli* at 100 μg/disc for a disc of 6 mm diameter (Table 3) [26]. Both 11-deoxyfistularin-3 (**1**) and dibromoverongiaquinol (**3**) showed strong antibacterial activities while 4,6-dibromo-2,5-dihydroxyphenylacetic acid methyl ester (**4a**) was inactive. Bromotyrosine metabolites have already been reported as antibacterials [7, 27–33].

In conclusion, three bromotyrosine metabolites **1**–**3** and the homogentisic acid derivative **4a** have been isolated from the marine sponge *Pseudoceratina* sp. collected in Vanuatu. 11-Deoxyfistularin-3 (**1**) and dibromoverongiaquinol (**3**) showed strong antibacterial activities against *S. aureus* and *E. coli* like several bromotyrosine metabolites isolated from marine sponges belonging to the order Verongida. Homogentisic acid derivative **4a** exhibited Pfnek-1 inhibitor activity (IC_50_ = 1.8 μM) and its unambiguous identification has been performed by synthesis. The study of the structure-activity relationships for the natural product **4a** and its synthetic analogues **4b**, **5**, **6a**, **6b**, **8**, **12**, **13a** and **13b** has highlighted the essential role of the bromine atoms and of the position of the hydroxyl groups for the inhibition of Pfnek-1. Consequently, the structural feature of homogentisic acid derivative **4a** could serve as a model for the development of new Pfnek-1 inhibitors. Replacement of the bromine atoms by other halogens (Cl, F) and the elongation of the ester chain could be particularly interesting to enhance the Pfnek-1 inhibition of these homogentisic acid derivatives. Finally, the moderate activity against *P. falciparum* could result from the weak capacity of the inhibitor to reach the kinase (or other) target(s), and demonstrates the difficulties met when using an enzymatic test for the screening of natural extracts and for the discovering of new anti-infectious drugs.

## 3. Experimental Section

### 3.1. Materials

The sponge of the genus *Pseudoceratina* Carter, 1885 (order Verongida, family Pseudoceratinidae) was collected by scuba diving at 40 m depth at Rowa islands, Banks Territory (Vanuatu). A voucher specimen, voucher number G318491, is deposited with the Queensland Museum, Brisbane, Australia. The FcB1 strain of *P. falciparum* was kindly provided by Dr. A. Valentin, Laboratory of Parasitology, Faculty of Pharmacy, Toulouse, France. Solvents were purchased from Ajax (Australia), and distilled before use, except for the HPLC grade methanol; Biochemical reagents were purchased from Sigma-Aldrich and Cambrex. Radioactive γ-[^33^P] ATP was purchased from Perkin Elmer (France). HPLC was performed on a Waters apparatus, (Waters 510 pumps; Waters 996 Photodiode Array Detector) using a μBondapack C_18_ column (125 Å, 10 μm, 4.6 × 250 mm). NMR spectra were recorded on Bruker AC 250, Bruker Avance 300, 400 and 500 spectrometers. Mass spectra were recorded on an ion trap LCQ Finnigan spectrometer in APCI ionization mode (positive or negative), except for compounds **13a** and **13b** (ESI ionization mode, positive). High resolution mass spectra were recorded on a GCT Premier apparatus (Waters Micromass) in CI (CH_4_) ionization mode. Radioactivity was measured using a liquid scintillation analyzer Packard Tri-carb 1600TR and Packard Ultima Gold MV scintillation cocktail.

### 3.2. Extraction and Isolation

The freeze-dried sponge was extracted twice overnight with fresh 95% EtOH at room temperature, filtered and the ethanol was evaporated. The residue was subjected to solvent partition between CH_2_Cl_2_ and H_2_O to give the CH_2_Cl_2_ soluble extract and the CH_2_Cl_2_/H_2_O insoluble extract.

The CH_2_Cl_2_ soluble extract (4 g) was subjected to silica gel column chromatography (Merck silica gel 60, 0.040–0.063 mm) using CH_2_Cl_2_/MeOH (98/2) as eluent to afford 12 fractions (F1-12). Fraction F11 (965 mg) was purified by semipreparative reversed-phase HPLC with H_2_O/MeOH gradient elution (t = 0: 40/60, t = 20 min: 25/75, t = 25 min: 0/100, t = 30 min: 40/60, flow rate: 3 mL/min, wavelength: 254 nm) to give 11-deoxyfistularin-3 (**2**; 13.6 mg, *t*_R_ = 12.8 min) and another fraction which was further purified by semipreparative reversed-phase HPLC with H_2_O/MeOH gradient elution (from t = 0 to t = 10 min: 80/20, t = 20 min: 0/100, t = 30 min: 80/20, flow rate: 3 mL/min, wavelength: 254 nm) to give dibromoverongiaquinol (**3**; 8.8 mg, *t*_R_ = 14.5 min).

The CH_2_Cl_2_/H_2_O insoluble extract (885 mg) was fractionated on silica gel by column chromatography (Merck silica gel 60, 0.040–0.063 mm) using CH_2_Cl_2_/MeOH (97/3) as eluant to afford seven fractions (F1-7). Fraction F5 (65 mg) was purified by Sep-Pak cartridge (silica, Waters) with *n*-hexane-AcOEt gradient elution to give methyl (2,4-dibromo-3,6-dihydroxyphenyl)acetate (**4a**, 24 mg). Finally, Fraction F7 (810 mg) was purified by preparative thick layer chromatography (CH_2_Cl_2_/MeOH: 97/3) to give 11,19-dideoxyfistularin-3 (**1**; 67 mg) and 11-deoxyfistularin-3 (**2**; 17 mg). The spectroscopic properties of the isolated tyrosine metabolites **1**–**3** were consistent with those previously published [18].

### 3.3. Protein Kinase Assay

A GST-Pfnek-1 fusion protein was purified from the *Escherichia coli* BL21 strain carrying a Pfnek-1 expression plasmid harbouring an ampicillin resistance cassette (bacteria kindly provided by D. Dorin, INSERM 511) as described by Dorin *et al*. [5]. Pfnek-1 kinase activity was determined by measuring the ^33^P incorporation in ß-caseine using γ[^33^P]-ATP.

Briefly, test compounds were dissolved in DMSO and diluted in 20 mM Tris, pH 7.5 20 mM MgCl_2_, 10 mM NaF and 10 mM ATP. β-casein (3 mg/mL) and γ[^33^P]-ATP were added prior to the addition of Pfnek-1 to start the kinase reaction. Approximately 5 μCi of γ[^33^P]-ATP was used per reaction. After incubation at 30 °C for 30 min, each solution was blotted on a phosphocellulose filter paper (P81 Whatman-cation exchange chromatography paper). After four washes with 1% H_3_PO_4_ at, the remaining radioactivity was measured using a liquid scintillation analyzer Packard 1600TR. The IC_50_ is defined as the concentration of compound which inhibits 50% of enzyme activity compared to the control (reaction in the absence of inhibitor).

### 3.4. Activity against Erythrocytic Stages of Cultured P. falciparum

The antiplasmodial activity was studied *in vitro* against the chloroquine-resistant *Plasmodium falciparum* strain FcB1 by a micromethod using the lactate deshydrogenase (LDH) assay (Makler and Hinrichs [34]). Parasites were cultivated using the method of Trager and Jensen [35]. Erythrocytes infected with *P. falciparum* (ring stage, 1% of parasitaemia) were re-suspended in complete culture medium at a haematocrit of 1.5%. The suspension was distributed in 96-well microtitre plates (200 μL per well). Drug testing was performed in triplicate. For each assay, a parasite culture was incubated with the drug for 48 h in 5% CO_2_ at 95% relative humidity, and frozen until the biochemical assay could be run. After defrosting, a 20 μL sub-sample of the contents of each well was mixed with 100 μL of a substrate solution containing 20 mg/mL of lithium l-lactate (Sigma), 5.5 mg/mL of TRIS (Sigma), and 3.7 mg/mL of 3-acetylpyridine adenine dinucleotide (APAD; Sigma), in the well of another microtitre plate. After incubation for 30 min, 25 μL of a mixture of NBT (1.6 mg/mL; Sigma) and PES (0.1 mg/mL; Sigma) were added to each well. After a further 35 min of incubation, the reaction was stopped by the addition of 25% acetic acid (25 μL per well). Accumulation of the reduced form of APAD was measured at λ = 650 nm, using a spectrophotometer (microplate reader, Metertech). IC_50_ values were determined graphically in a concentration versus percent inhibition curve.

### 3.5. Synthesis

#### 4,6-Dibromo-5-hydroxy-3H-benzofuran-2-one (**6a**) and 6-bromo-5-hydroxy-3H-benzofuran-2-one (**6b**)

5-Hydroxy-*3H*-benzofuran-2-one (**5**, 0.20 g, 1.33 mM), *N*-bromosuccinimide (0.52 g, 2.93 mM) and *p*-toluenesulfonic acid (0.30 g, 0.13 mM) were added to dichloromethane (20 mL) and stirred for 12 h at room temperature. The solution was evaporated under vacuum. The residue was diluted with water and extracted with ethyl acetate. The organic layer was washed with brine, dried over anhydrous sodium sulfate, and concentrated under vacuum. The residue was purified on silica gel by column chromatography (ethyl acetate/hexane: 1/1) to give **6a** (86 mg, 21%, white powder) and **6b** (37 mg, 12%, white powder). Compound **6a**: ^1^H-NMR [300 MHz, (CD_3_)_2_SO)] δ 9.89 (s, 1H), 7.47 (s, 1H), 3.84 (s, 2H). ^13^C-NMR [75 MHz, (CD_3_)_2_SO]: δ 173.2, 147.7, 147.6, 127.1, 113.9, 111.1, 109.5, 35.6. APCI-MS : 305, 307, 309 [M-H]^−^; Compound **6b**: ^1^H-NMR [300 MHz, (CD_3_)_2_SO]: δ 10.09 (s, 1H), 7.36 (s, 1H), 6.95 (s,1H), 3.84 (s, 2H). ^13^C-NMR [75 MHz, (CD_3_)_2_SO]: δ 174.9, 151.0, 147.4, 125.3, 114.5, 112.9, 107.8, 33.6; APCI-MS : 227, 229 [M-H]^−^.

#### Methyl (2,4-dibromo-3,6-dihydroxyphenyl)acetate (**4a**)

4,6-Dibromo-5-hydroxy-*3H*-benzofuran-2-one (**6a**, 0.14 g, 0.46 mM) and *p*-toluenesulfonic acid (0.17 g, 0.92 mM) were added to methanol (50 mL) and stirred for 24 h at room temperature. The solution was evaporated under vacuum. The residue was diluted with water and extracted with ethyl acetate. The organic layer was washed with brine, dried over anhydrous sodium sulfate, and concentrated under vacuum. The residue was purified on silica gel by column chromatography (ethyl acetate/hexane: 1/1) and **4a** was obtained (131 mg, 84%) as a white powder. ^1^H-NMR (300 MHz, CD_3_OD): δ 7.00 (s, 1H), 3.85 (s, 2H), 3.70 (s, 3H); ^13^C-NMR (75 MHz, CD_3_OD): δ 171.6, 149.8, 143.8, 122.2, 117.3, 115.7, 109.7, 51.1, 35.0; APCI-MS : 337, 339, 341 [M-H]^−^.

#### Methyl (4-bromo-3,6-dihydroxyphenyl)acetate (**4b**)

The above mentioned protocol was used to give **4b** (46 mg, 78%) as a white powder from **6b** (52 mg). ^1^H-NMR (300 MHz, CD_3_OD): δ 6.84 (s, 1H), 6.65 (s, 1H), 3.61 (s, 3H), 3.46 (s, 2H); ^13^C-NMR (75 MHz, CD_3_OD): δ 176.6, 152.7, 150.5, 125.6, 122.3, 121.5, 111.7, 54.9, 39.0; APCI-MS: 258, 260 [M-2H] ^−^, 259, 261 [M-H]^−^; HRMS (CI, CH_4_): 259.9675 (Calc. for C_9_H_9_O_4_^79^Br, [M]^−^, 259.9684).

#### Methyl (2,5-dihydroxyphenyl)acetate (**8**)

Homogentisic acid **7** (2 g, 11.89 mM) and concentrated hydrochloric acid (1 mL) were added to methanol (100 mL) and stirred for 24 h at room temperature. The solution was evaporated under vacuum. The residue was diluted with water and extracted with ethyl acetate. The organic layer was washed with brine, dried over anhydrous sodium sulfate, and concentrated under vacuum. The residue was purified on silica gel by column chromatography (dichloromethane) and **8** was thus obtained (2.1 g, 97%) as a white powder. ^1^H-NMR [300 MHz, (CD_3_)_2_SO]: δ 8.73 (s,1H), 8.63 (s,1H), 6.60–6.46 (m, 3H), 3.58 (s, 3H), 3.47 (s, 2H); ^13^C-NMR [75 MHz, (CD_3_)_2_SO]: δ 172.2, 150.0, 148.2, 122.0, 117.9, 115.8, 114.7, 51.8, 35.5; APCI-MS: 181 [M-H]^−^.

#### 2′,4′-Dibenzyloxyacetophenone (**10**)

2′,4′-Dihydroxyacetophenone (**9**, 20.0 g, 131.5 mM), potassium carbonate (46.0 g, 332.8 mM) and benzyl bromide (47.2 g, 276.0 mM) were added to acetone (150 mL) and stirred for 15 h at room temperature. The solution was filtered and evaporated under vacuum. The residue was purified by recrystallization from hexane to give **10** (31.9 g, 73%) as a white powder. ^1^H-NMR (300 MHz, CDCl_3_): δ 7.85 (d, *J* = 9.3 Hz, 1H), 7.35–7.43 (m, 10H), 6.62 (dd, *J* = 9.3 Hz, *J* = 2.6 Hz, 1H), 6.61 (d, *J* = 2.7 Hz, 1H), 5.12 (s, 2H), 5.09 (s, 2H), 2.56 (s, 3H); ^13^C-NMR (75 MHz, CDCl_3_): δ 197.7, 163.5, 160.1, 136.2, 136.0, 132.7, 128.7–127.6, 121.7, 106.4, 100.4, 99.9, 70.7, 70.3; APCI-MS: 355 [M+Na]^+^, 687 [2M+Na]^+^.

#### Methyl (2,4-dihydroxyphenyl)acetate (**11**)

2′,4′-Dibenzyloxyacetophenone (**10**, 340 mg, 1.025 mM), perchloric acid (0.5 mL) and thallium trinitrate (466 mg, 1.05 mM) were added to tetrahydrofuran/methanol (1/5, 3 mL) and stirred for 18 h at room temperature. The solution was filtered and the residue was washed with methanol. The residue was diluted with water and extracted with ethyl acetate. The organic layer was washed with brine, dried over anhydrous sodium sulfate, and concentrated under vacuum. Without further purification, the mixture (278 mg), palladium 10% on carbon (190 mg) and ammonium formate 97% (331 mg, 5.25 mM) were added to methanol (12 mL) and stirred for 4 h at room temperature. The mixture was filtered over celite and the filtrate evaporated under vacuum. The residue was diluted with water and extracted with ethyl acetate. The organic layer was washed with brine, dried over anhydrous sodium sulfate, and concentrated under vacuum. The residue was purified on silica gel by column chromatography (methanol/dichloromethane: 2/98) and **11** was thus obtained (95 mg, 51%, two steps) as a white powder. ^1^H-NMR (300 MHz, CDCl_3_): δ 8.42 (s, 2H), 6.94 (d, *J* = 8.2 Hz, 1H), 6.43 (d, *J* = 2.4 Hz, 1H), 6.39 (dd, *J* = 8.2 Hz, *J* = 2.4 Hz, 1H), 3.79 (s, 3H), 3.62 (s, 2H); APCI-MS: 181 [M-H]^−^.

#### Methyl (3,5-dibromo-2,4-dihydroxyphenyl)acetate (**12**)

Methyl (2,4-dihydroxyphenyl)acetate (**11**, 1.50 g, 8.23 mM), *N*-bromosuccinimide (3.08 g, 17.29 mM) and *p*-toluenesulfonic acid (0.16 g, 0.82 mM) were added to methanol (80 mL) and stirred for 3 h at 40 °C in an ultrasonic bath. The solution was evaporated under vacuum. The residue was diluted with water and extracted with ethyl acetate. The organic layer was washed with brine, dried over anhydrous sodium sulfate, and concentrated under vacuum. The residue was purified on silica gel by column chromatography (ethyl acetate/hexane: 1/1) and **12** was thus obtained (2.04 g, 73%) as a white powder. ^1^H-NMR [400 MHz, (CD_3_)_2_SO]: δ 9.61 (s, 1H), 9.31 (s, 1H), 7.32 (s, 1H), 3.61 (s, 2H), 3.59 (s, 3H); ^13^C-NMR [100 MHz, (CD_3_)_2_SO]: δ 172.0, 153.2, 151.0, 133.4, 117.5, 103.3, 101.3, 52.3, 35.5; ^1^H-NMR [300 MHz, CD_3_OD]: δ 7.35 (s, 1H), 3.77 (s, 3H), 3.69 (s, 2H); ^13^C-NMR [75 MHz, CD_3_OD]: δ 174.1, 153.9, 152.0, 133.9, 117.6, 102.2, 101.2, 52.5, 36.3; APCI-MS: 337, 339, 341 [M-H]^−^; HRMS (CI, CH_4_): 337.8787 (Calc. for C_9_H_8_O_4_^79^Br_2_, [M]^−^, 337.8789).

#### Methyl (3,6-dibenzoyl-2,4-dibromophenyl)acetate (**13a**)

Methyl (2,4-dibromo-3,6-dihydroxyphenyl)acetate (**4a**, 20 mg, 0.0588 mM) was dissolved in dichloromethane (1mL). At 0 °C, benzoyl chloride (14.3 μL, 0.0123 mM) and triethylamine (14.1 μL, 0.123 mM) were added. After 1 hour at 0 °C, water (5 mL) and dichloromethane (5 mL) were added. The organic layer was separated, dried over anhydrous sodium sulfate, and concentrated under vacuum. The residue was purified on silica gel by column chromatography (cyclohexane, then 50/50 cyclohexane/CH_2_Cl_2_) to give **13a** (23 mg, 70%) as a colorless oil. ^1^H-NMR (300 MHz, CDCl_3_): δ 8.32–7.54 (m, 11H), 3.92 (s, 2H), 3.65 (s, 3H); ^13^C-NMR (75 MHz, CDCl_3_): δ 169.3, 163.9, 162.9, 147.6, 144.7, 134.2, 134.1, 130.6, 130.3, 128.8, 128.7, 128.6, 128.3, 126.3, 121.6, 116.4, 52.3, 36.6 ESI-MS: 569, 571, 573 [M+Na]^+^, 585, 587, 589 [M+K]^+^; HRMS (CI, CH_4_): 545.9308 (Calc. for C_23_H_16_O_6_^79^Br_2_, [M]^−^, 545.9314).

#### Methyl (3,6-dibenzoyl-4-bromophenyl)acetate **13b**

The above mentioned protocol was used to give **13b** from **4b** with a similar yield. ^1^H-NMR (300 MHz, CDCl_3_): δ 8.29–7.38 (m, 12H), 3.66 (s, 2H), 3.63 (s, 3H); ^13^C-NMR (75 MHz, DCl_3_): δ 170.1, 164.2, 164.1, 147.0, 146.1, 134.0, 134.9, 130.4, 130.2, 128.8, 128.7, 128.6, 128.3, 127.4, 127.3, 125.8, 115.1, 52.2, 35.9; ESI-MS: 491,493 [M+Na]^+^, 507, 509 [M+K]^+^, HRMS (CI, CH_4_): 468.0208 (Calc. for C_23_H_17_O_6_^79^Br, [M]^−^, 468.0208).

## Figures and Tables

**Figure 1.**
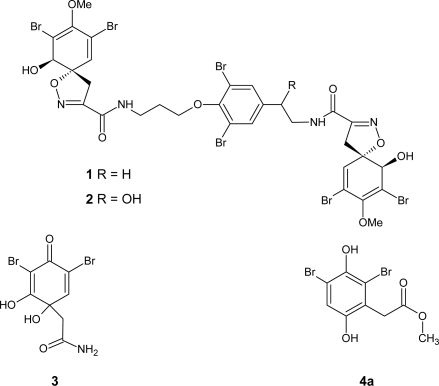
Bromotyrosine metabolites **1**–**3** and methyl (2,4-dibromo-3,6-dihydroxyphenyl) acetate (**4a**).

**Table 1.** Comparison of NMR data between natural and synthetic compound **4a**.

	**^1^H, CD_3_OD, 300 MHz**			**^13^C, CD_3_OD^1^**								
**4a** synthetic	7.01	3.86	3.70	171.6	149.8	143.8	122.2	117.3	115.7	109.7	51.1	35.0
**4a** natural	6.88	3.73	3.57	171.6	149.8	143.8	122.2	117.3	115.6	109.7	51.1	35.0

1 The ^13^C-NMR spectrum of the natural compound was recorded at 75 MHz; the ^13^C-NMR spectrum of the synthetic compound was recorded at 125 MHz.

**Table 2.** Pfnek-1 inhibitory and *P. falciparum* activities of compounds **1**–**6, 8, 12**–**13** (IC_50_ values are in μM).

**Compound number**	**Pfnek-1**	**FCB1 of *P. falciparum***
**1**	>50	>100
**2**	>50	>100
**3**	>50	>100
**4a**	1,8	12
**4b**	10	26
**5**	>50	29
**6a**	>50	35
**6b**	>50	17
**8**	>50	36
**12**	>50	>100
**13a**	>50	22.5
**13b**	>50	13

**Table 3.** Antibacterial activity of natural products **1**–**3** and **4a**.

**Compound number^1^**	**Growth inhibition diameter (mm)**	
	***S. aureus***	***E. coli***
**1**	14	16
**2**	0	0
**3**	20	15
**4a**	0	0
gentamycin	16	-
chloramphenicol	-	17

1 Compounds **1–3**, **4a** were evaluated at 100 μg/disc, gentamicine at 10 μg/disc and chloramphenicol at 30 μg/disc.
